# Italian Association of Clinical Endocrinologists (AME) and Italian Chapter of the American Association of Clinical Endocrinologists (AACE) Position Statement: Clinical Management of Vitamin D Deficiency in Adults

**DOI:** 10.3390/nu10050546

**Published:** 2018-04-27

**Authors:** Roberto Cesareo, Roberto Attanasio, Marco Caputo, Roberto Castello, Iacopo Chiodini, Alberto Falchetti, Rinaldo Guglielmi, Enrico Papini, Assunta Santonati, Alfredo Scillitani, Vincenzo Toscano, Vincenzo Triggiani, Fabio Vescini, Michele Zini, on behalf of AME and Italian AACE Chapter

**Affiliations:** 1 Department of Internal Medicine, “S. M. Goretti” Hospital, 04100 Latina, Italy; 2 Endocrinology Service, Galeazzi Institute IRCCS, 20161 Milan, Italy; roberto.serena@libero.it; 3 Ospedale Classificato Villa Salus, 30174 Venezia Mestre, Italy; cptmrc@gmail.com; 4 General Medicine and Endocrinology, University Hospital, 37126 Verona, Italy; roberto.castello@aovr.veneto.it; 5 Unit for Bone Metabolism Diseases and Diabetes & Lab of Endocrine and Metabolic Research, IRCCS Istituto Auxologico Italiano, 20149 Milan, Italy; iacopo.chiodini@unimi.it; 6 Department of Clinical Sciences and Community Health, University of Milan, 20149 Milan, Italy; 7 Centro Hercolani and Villa Alba (GVM), 40123 Bologna and EndOsMet, Villa Donatello Private Hospital, 50132 Florence, Italy; alberto.falchetti2@alice.it; 8 Department of Endocrinology and Metabolic Diseases, Regina Apostolorum Hospital, Albano Laziale, 00041 Rome, Italy; rinaldo.guglielmi@gmail.com (R.G.); papinie@gmail.com (E.P.); 9 Department of Endocrinology and Diabetes, San Giovanni Addolorata Hospital, 00184 Rome, Italy; a.santonati1@fastwebnet.it; 10 Endocrinology Unit, Department of Medical Science, Ospedale Casa Sollievo della Sofferenza IRCCS, 71013 San Giovanni Rotondo (FG), Italy; alscill@tin.it; 11 Endocrinology, Department of Clinical and Molecular Medicine, Sant‘Andrea Hospital, Sapienza University of Rome, 00189 Roma, Italy; vincenzo.toscano@uniroma1.it; 12 Interdisciplinary Department of Medicine, Endocrinology and Metabolic Diseases, University of Bari “Aldo Moro”, 70124 Bari, Italy; vincenzo.triggiani@uniba.it; 13 Department of Endocrinology and Diabetes, Santa Maria della Misericordia Hospital, 33010 Udine, Italy; fabio.vescini@asuiud.sanita.fvg.it; 14 Endocrinology Unit, Arcispedale S. Maria Nuova IRCCS, 42123 Reggio Emilia, Italy; michele.zini@ausl.re.it

**Keywords:** Vitamin D, cholecalciferol, ergocalciferol, calcifediol, calcitriol, bone

## Abstract

Vitamin D deficiency is very common and prescriptions of both assay and supplementation are increasing more and more. Health expenditure is exponentially increasing, thus it is timely and appropriate to establish rules. The Italian Association of Clinical Endocrinologists appointed a task force to review literature about vitamin D deficiency in adults. Four topics were identified as worthy for the practicing clinicians. For each topic recommendations based on scientific evidence and clinical practice were issued according to the Grading of Recommendations, Assessment, Development, and Evaluation (GRADE) System. (1) What cut-off defines vitamin D deficiency: even though 20 ng/mL (50 nmol/L) can be considered appropriate in the general population, we recommend to maintain levels above 30 ng/mL (75 nmol/L) in categories at risk. (2) Whom, when, and how to perform screening for vitamin D deficiency: categories at risk (patients with bone, liver, kidney diseases, obesity, malabsorption, during pregnancy and lactation, some elderly) but not healthy people should be screened by the 25-hydroxy-vitamin D assay. (3) Whom and how to treat vitamin D deficiency: beyond healthy lifestyle (mostly sun exposure), we recommend oral vitamin D (vitamin D2 or vitamin D3) supplementation in patients treated with bone active drugs and in those with demonstrated deficiency. Dosages, molecules and modalities of administration can be profitably individually tailored. (4) How to monitor the efficacy of treatment with vitamin D: no routine monitoring is suggested during vitamin D treatment due to its large therapeutic index. In particular conditions, 25-hydroxy-vitamin D can be assayed after at least a 6-month treatment. We are confident that this document will help practicing clinicians in their daily clinical practice.

## 1. Introduction

Vitamin D3 (cholecalciferol) is produced in the skin from 7-dehydrocholesterol by ultraviolet (UV) radiations at levels of sunlight exposure that do not induce skin burns (UV 290–315 nm) and is subsequently removed after its binding to vitamin D-binding protein (VDBP). Liver and other body tissues metabolize vitamin D3 synthesized in the skin and the orally ingested vitamin D2 (ergocalciferol) and D3 to 25-hydroxy-vitamin D [25(OH)D], the main circulating form, by means of 25-hydroxylase activity; 25(OH)D is then further metabolized in the kidneys to 1,25-dihydroxyvitamin D [1.25(OH)_2_D] by the enzyme CYP27B1 to regulate calcium, phosphate and bone metabolism. In addition, a wide variety of non-calcemic tissues and cells, including macrophages, also convert 25(OH)D to [1.25(OH)_2_D] for the purpose of regulating a variety of biologic functions in an autocrine/paracrine manner; 1.25(OH)_2_D is the major hormonal form of vitamin D and is responsible for most of its biologic actions. The tightly controlled production of 1.25(OH)_2_D in the kidneys is stimulated by the parathyroid hormone (PTH) and is inhibited by calcium, phosphate and fibroblast growth factor (FGF)-23 [1].

Vitamin D metabolites are transported in the blood bound to VDBP and albumin, produced by liver, and only a minority circulates as a free form. The receptors for 1.25(OH)_2_D (vitamin D receptor, VDR) are widely distributed transcription factors that regulate the expression of the genes, which mediate its biologic activity [1].

The classic target tissues—bone, gut, and kidney—are involved with calcium homeostasis, mainly through the regulation of transcellular calcium transport; 1.25(OH)_2_D stimulates calcium absorption in the intestine and calcium reabsorption in the distal tubule of the kidney; 1.25(OH)_2_D regulates both the formation and resorption of bone by promoting the differentiation of osteoblasts and regulating the production of proteins such as collagen, alkaline phosphatase (ALP), osteocalcin and Receptor Activator of Nuclear Factor-Kappa B Ligand (RANKL) [1].

Subclinical deficiency of vitamin D is a highly prevalent condition in the general population and, in recent years, an increasing number of subjects are treated with different formulations of vitamin D, thus increasing costs linked to vitamin D assays and preparations.

Presently, physicians involved in the prescription of vitamin D demonstrate a variable clinical approach to the screening of its deficiency, the modalities of treatment and the monitoring of therapy over time. In order to overcome these shortcomings, the Italian Association of Clinical Endocrinologists (AME) appointed, in 2016, a panel of experts for the definition of the optimal management of vitamin deficiency in clinical practice.

During a preliminary symposium, 200 physicians with specific expertise discussed the relevant items and identified the main hot topics in vitamin D deficiency. Subsequently, the expert panel reviewed the pertinent literature data and achieved a consensus on the recommendations. Whenever evidence was contrasting or equivocal, a majority of 2/3 was required to provide conclusions that were based on the panelists‘ clinical experience.

The Grading of Recommendations, Assessment, Development, and Evaluation (GRADE) system was adopted for the present Position Statement [2,3,4]. According to GRADE, evidence is categorized into four quality levels (high, moderate, low, or very low), while recommendations are classified as strong (“recommendations”) or weak (“suggestions”), on the basis of the quality of supporting evidence and level of agreement between the panel members [3]. Whenever possible, the level of evidence (LoE) has been reported beside each quotation using the following symbols: very low (⊗◯◯◯), low (⊗⊗◯◯), moderate (⊗⊗⊗◯) and high (⊗⊗⊗⊗) quality (Appendix Table A1). Briefly, “very low quality” evidence is derived from unsystematic clinical observations (case report, case series) or very indirect evidence (i.e., surrogate end-points); “low quality” evidence is from observational studies or randomized controlled trials (RCT) with major limits; “moderate quality evidence” derives from RCTs with important limitations or from rigorous observational studies; “high quality evidence” are well performed RCTs and, exceptionally, strong evidence from unbiased observational studies [3].

As recently underlined [5], further research is needed to inform better clinical guidelines in this area, and to assess implementation practices that will encourage evidence-based management practices for vitamin D insufficiency in adult populations. Moreover, greater understanding of physician management of uncertainty in clinical practice may help to avoid overutilization and inconsistent practice in similar clinical situations.

## 2. Clinical Issues

### 2.1. What Is the Cut-Off That Defines Vitamin D Deficiency?

As the plasma 25(OH)D levels are regarded as the most reliable indicator of vitamin D storage in the human body [1,6], the diagnosis of vitamin D deficiency is based on the determination of total plasma 25(OH)D concentrations.

At present, there is no agreement on “normal levels” of 25(OH)D. Throughout the years, the cut-off was progressively increased from 12 to 20 and, finally, to 30 ng/mL (30, 50, 75 nmol/L, respectively), mainly because of the confusion of the normal with the desirable levels [7,8]. Normal levels are defined as those between ±2 standard deviation (SD) from the mean values in normal population, while desirable levels are set by regulatory agencies for the prevention of diseases on the basis of observational studies.

Currently, there is consensus that 25(OH)D levels lower than 20 ng/mL (50 nmol/L) are associated in adults with:

- Secondary hyperparathyroidism, osteomalacia or osteoporosis [9,10,11];
- Proximal limb muscle weakness, ataxia, and increased risk of falls [12,13];
- Increased risk of fractures [14];
- Hampered effect of drugs used for osteoporosis [15].

In 2010, the Institute of Medicine (IOM), due to the lack of evidence for a benefit derived from increase in the normal threshold of vitamin D, defined deficiency, insufficiency and sufficiency of 25(OH)D as a serum value <12 ng/mL (30 nmol/L), 12–20 ng/mL (30–50 nmol/L) and 20–30 ng/mL (50–75 nmol/L), respectively [16].

In 2011 the USA Endocrine Society together with other Scientific Societies upgraded these three thresholds to <20 ng/mL (50 nmol/L), 20–30 ng/mL (50–75 nmol/L), and 30–100 ng/mL (75–250 nmol/L), respectively [6]. The upgrading was based on the demonstration (even if with a low quality evidence) of an increased intestinal calcium absorption [17] and a decreased level of circulating PTH (reviewed in 6) when 25(OH)D values were >30 ng/mL (75 nmol/L). These higher cut-offs were also based on the variability of the available 25(OH)D assay results and on the results of an autopsy study on subjects dead after traffic accidents [18]. Histomorphometric evaluation showed large osteoid areas (allegedly corresponding to vitamin D deficiency) mostly in presence of 25(OH)D levels < 20 ng/mL (50 nmol/L) or, less frequently (namely 21%) between 20 and 30 ng/mL (50 and 75 nmol/L). That study of course did not employ the gold standard method of double tetracycline labeling and is further biased by the lack of data about renal function, calcium levels and intake, physical activity, and so on.

Notably, seasonal variations in vitamin D plasma levels are well established, with values that are higher in Summer and Autumn than in Winter and Spring [19]. The latitude (northern vs. southern), skin color (black vs. white), sex (females vs. males), and body mass index (BMI) (higher vs. lower) contribute to the variability of serum vitamin D [20,21], as well.

An extensive revision of the data regarding the 25(OH)D target levels for the different outcomes is not the aim of the present position statement. For cancer prevention, however, serum 25(OH)D levels between 36 and 48 ng/mL are reportedly associated with favorable outcomes. For the improvement of endpoints such as bone mineral density (BMD), lower extremity function, dental health, incident falls, fractures, hypertension and admission to nursing home, the most appropriate serum 25(OH)D level is described as greater than 30 ng/mL (75 nmol/L) [22]. These cut-offs, however, are not supported by high quality evidence.

Literature data are thus univocal for the indication to vitamin D treatment in all subjects with serum 25(OH)D levels < 20 ng/mL (50 nmol/L) but are controversial for values between 20 and 30 ng/mL (50 and 75 nmol/L). The revision of the major meta-analyses of the RCTs on vitamin D supplementation was not, similarly to cancer prevention, one of the aims of this position statement. However, the experts‘ panel agreed that, even if available data are not consistent, a serum 25(OH)D level of at least 30 ng/mL (75 nmol/L) should be the target for the prevention of fracture risk and muscle function deterioration in older adults [22]. So, when 25(OH)D values are between 20 and 30 ng/mL (50 and 75 nmol/L), particularly if they are measured in Summer/Autumn, the measurement of serum PTH may be used for the confirmation of an actual vitamin D deficiency [23]. The relationship between 25(OH)D and PTH values, however, is not linear, it depends even on calcium intake [24], the threshold for plateau is not clearly defined [9,16,25], and secondary hyperparathyroidism reportedly occurred only in a third of patients of a large series with 25(OH)D values ≤ 12 ng/mL (30 nmol/L) [26].


**We recommend** to maintain 25(OH)D levels above 30 ng/mL (75 nmol/L) in subjects:
With osteopenia, osteoporosis or fragility fractures;On treatment for osteoporosis;Who belong to at risk categories (see Section 3.1).**We suggest** to consider serum PTH measurement when vitamin D values are lower than 30 ng/mL (75 nmol/L), particularly if tested in Summer and Autumn.


### 2.2. What Is the Scope of the Problem?

The estimated prevalence of vitamin D deficiency in adult population depends on its cut-off definition (specifically, <20 vs. <30 ng/mL) [10,11]. The National Health and Nutrition Examination Survey (NHANES, 2001–2006) showed that 25% of population was at risk for insufficiency, as defined by serum 25(OH)D levels of 12 to 20 ng/mL, and that 8% had very low 25(OH)D levels (<12 ng/mL) [27].

In NHANES, mean 25(OH)D levels appeared lower in the years 2000–2004 than in 1988–1994, but these changes are due to assay changes rather than to an actual decline. In an adult subgroup from NHANES, however, changes in BMI, milk intake, and sun protection appeared to contribute to a small but real decline in vitamin D status [27]. If the 25(OH)D cut-off value for the definition of vitamin D deficiency is raised to 30 ng/mL, its prevalence obviously increases. According to NHANES data (2001 to 2006), 42% of subjects showed 25(OH)D levels between 20 and 30 ng/mL [27] and were classified as affected by vitamin D insufficiency according to Endocrine Society guidelines [6].

In a study on Swedish healthy people, approximately 75% of the subjects had serum 25(OH)D values < 30 ng/mL (<75 nmol/L) during 75% of the year and 50% had serum 25(OH)D < 20 ng/mL (<50 nmol/L) during 50% of the year [28]. In Switzerland, the prevalence of vitamin D insufficiency (serum 25(OH)D levels between 20 ng/mL and 30 ng/mL, 50 nmol/L and 75 nmol/L) and deficiency (<20 ng/mL, <50 nmol/L) was 36% and 38%, respectively [29]. In spite of the much lower latitude, a study from Turkey reported serum 25(OH)D values lower than 30 ng/mL (75 nmol/L) in 75% of the cases [30].

As for Italy, several studies on vitamin D status have been performed over the past 20 years in populations that embrace different age ranges and living conditions. Isaia et al. reported 25(OH)D circulating levels less than 12 ng/mL (30 nmol/L) in 76% of Italian women over 70 years of age, in late Winter [31]. In subjects institutionalized or with underlying diseases, the percentage of subjects with hypovitaminosis D was even more. Moreover, in 608 young and healthy women, 30% resulted to be deficient (cut-off of serum 25(OH)D < 20 ng/mL, 50 nmol/L) [32]. In younger subjects also, the levels of vitamin D were lower in women and in Winter [33]. The InChianti studio, that from 1998 studies the aging processes on 1107 participants and collects information about diet, sun exposure, disability, kidney function, levels of 25(OH)D and PTH, revealed values of serum vitamin D, on average, higher than 20 ng/mL (50 nmol/L) in healthy adults, but significantly reduced in males ≥ 60 years and females ≥ 50 years [34,35]. In the study by Houston et al., 64% of subjects > 65 years (average 75 years) had values < 20 ng/mL (50 nmol/L), and deficiency of vitamin D was found in approximately 30% of women and 14% of males and insufficiency was in 75% of women and 51% of males [36]. Another Italian study on 974 patients, ≥75 years of age, with femoral fracture from 4 large provincial hospitals, located in Central-Northern Italy, showed 25(OH)D circulating mean levels of 12.2 ± 9.4 ng/mL (30.5 ± 23.5 nmol/L) with >50% having <12 ng/mL (30 nmol/L) and only 16% > 20 ng/mL (50 nmol/L) [37]. A recent observational study on serum vitamin D levels in Italian pediatric populations/young adults demonstrated 25(OH)D levels not significantly reduced in the young and healthy young adults. In 113 normal weight and 444 obese children (prepubertal and pubertal), approximately 70% of normal weight children had 25(OH)D levels > 30 ng/mL (75 nmol/L) and 30% < 30 ng/mL, and approximately 55% of obese children had 25(OH)D levels > 30 ng/mL and 45% < 30 ng/mL [38]. Another pediatric Italian study revealed 50% of teenagers with 25(OH)D levels >30 ng/mL (75 nmol/L). Logistic regression analysis showed the following odd ratios (OR) in these specific dichotomous categories: overweight (OR 5.02) and obese (OR 5.36) versus subjects with normal BMI, lack of sun exposure (OR 8.64) versus optimal, regular use of “sunscreens” (OR 7.06) versus non-regular users. Moreover, significant higher relative risk for hypovitaminosis D was observed in Winter (OR 27.20), Spring (OR 26.44), Fall (OR 8.27) versus Summer [39].

So, the available data in Italy confirm unequivocally high prevalence of vitamin D deficiency in the elderly, especially in certain subgroups at greater risk and therefore, different strategies, according to the age groups in which no deficiency is generalized (strategies of “case finding”), and elderly population in which an overall situation of deficiency is expected, are necessary.

### 2.3. Vitamin D Deficiency and Damage to Organs Beyond Bone

Several reports show an association of vitamin D deficiency with increased risk of mortality [40], cancer (particularly colon, prostate, and breast cancer) [41], cardiovascular disease [42], type 1 and type 2 diabetes [43], autoimmune diseases [44], and decreased fertility [45].

Even though the topic is beyond the scope of our statement, we did not find evidence-based data demonstrating the efficacy of vitamin D for decreasing the risk of these chronic diseases [46,47]. Accordingly, we believe that vitamin D determination and substitution treatment are not yet warranted to prevent or treat clinical disorders that are different from bone diseases.

## 3. Diagnostic Issues

### 3.1. When to Order a Vitamin D Assay?

Individuals at risk should always be screened for vitamin D deficiency (Table 1). As in these subjects vitamin D treatment is expected to produce a rapid favorable effect, vitamin D determination is definitely cost-effective [6,29,48,49].

Even though many authors found low vitamin D plasma levels in healthy people worldwide, no evidence exists as for a benefit of vitamin D deficiency screening and/or treatment at a general population level [6].


**We recommend** screening for vitamin D deficiency in at risk populations.**We recommend against** screening for vitamin D deficiency in healthy people.


### 3.2. Which Molecular Forms of Vitamin D Are Assayed by Laboratories?

The vast majority of clinical laboratories measure circulating serum 25(OH)D that is the sum of 25(OH) vitamin D2 plus 25(OH) vitamin D3 [50].

Total serum 25(OH)D is the best available indicator of cutaneous synthesis (sunlight, skin) and total intake (food, supplements). Due to the widespread use of both vitamin D2 and vitamin D3 supplements, assays should always measure both 25(OH)D2 and 25(OH)D3, which is not the case for some immunoassays. To accurately measure total vitamin D, it must be dissociated from VDBP: in automated methods, details of this process are generally proprietary to the assay platform, and not known. Other possible explanations for different results by various immunoassays are:

- Cross reactivity with metabolites (such as 3-epi 25OH-D) that is variable in different kits.
- Presence of eterophilic antibodies.
- Lack of assay standardization.

To minimize these drawbacks, the standardization of 25(OH)D values by immunoassay methods to liquid chromatography (LC)–Tandem mass spectrometry (MS/MS) equivalent values or direct measurement by LC–MS/MS will provide valid conclusions about the actual health implications of vitamin D deficiency or insufficiency [51,52].


**We suggest** to employ the same method for serial measurements of vitamin D in any patient (panel agreed on the recommendation and downgraded it to suggestion due to feasibility reasons).


### 3.3. 1,25-(OH)_2_-Vitamin D Assay: Friend or Foe?

Serum levels of 1.25(OH)_2_D have little or no relationship to vitamin D stores. They are primarily regulated by PTH and FGF-23 levels, which, in turn, are regulated by calcium, vitamin D and phosphates [48,53]. So, in vitamin D deficiency 1.25(OH)_2_D levels increase and confusion may arise if its blood concentration is assumed as a measure of vitamin D storage [54].

1.25(OH)_2_D determination may be useful in a few clinical conditions [55,56,57]:

- When an elevated calcemia is associated with a low PTH level, as in granulomatous diseases (tuberculosis, sarcoidosis) and in some lymphomas.
- In some patients with end-stage kidney disease.
- In hereditary or acquired disorders of vitamin D and phosphate metabolism.

**We recommend against** routine 1,25-(OH)_2_-vitamin D assessment.


### 3.4. Which Additional Parameters May Be Useful in an Incidental Finding of Vitamin D Deficiency?

In case of an incidental finding of very low 25(OH)D levels, serum calcium, phosphate, ALP, PTH, magnesium, and creatinine should be evaluated [58,59,60]. These data better define the repletion of body stores and may suggest the screening for potentially concomitant low vitamin D-associated diseases (see Table 1 for possible differential diagnoses).


**We suggest** the evaluation of the above-mentioned laboratory parameters in selected cases, specifically for the screening of potentially concomitant low vitamin D-associated diseases.


### 3.5. Should a Severe Vitamin D Deficiency Lead to Dual Energy X-ray Absorptiometry (DXA) Evaluation?

Vitamin D deficiency may cause osteomalacia and increase the risk of low bone mass and fragility fractures [14]. It is appropriate to perform a densitometric evaluation by DXA at spine and hip in any subject whose risk of fractures is increased. Briefly, BMD testing is indicated in women aged 65 and in men aged 70 and older, in post-menopausal women younger than 65 and men < 70 years with low body weight, with prior fractures, who take drugs associated with bone loss, or with diseases or conditions associated with bone loss. All patients considered for a pharmacologic therapy for osteoporosis should first receive BMD testing [61]. The presence of asymptomatic fractures should be ruled out, also considering the familiarity for fragility fractures.


**We suggest** to perform DXA examination whenever the fracture risk is increased.


### 3.6. Vitamin D Should Be Checked after a Fragility Fracture?

As vitamin D insufficiency/deficiency may impair the response to the bone-active drugs [15], it appears reasonable to test vitamin D levels previously to the treatment with any bone-active drug, and, if necessary, to assure adequate supplementation.


**We suggest** to check 25(OH)D levels in any patient with established osteoporosis before starting the treatment.


### 3.7. How to Manage the Persistence of Severe Vitamin D Deficiency after Loading Doses and Chronic Replacement Therapy?

Instead of further increasing vitamin D dosage, it is appropriate to rule out secondary causes of vitamin D deficiency by lab assays as depicted at Section 3.4. Other procedures may be considered according to clinical context [62].


**We recommend** to rule out secondary causes of vitamin D deficiency whenever serum 25(OH)D levels are not normalized as expected after treatment.


## 4. Therapeutic Issues

### 4.1. Fortified Food for Treating Vitamin D Deficiency: What Is Their Role?

The Dietary Guidelines for Americans identified vitamin D among the four foods of interest for public health [63].

The major dietary source of vitamin D is cod liver oil, but also fishes, such as mackerel, carp, eel, salmon, smoked sturgeon, trout, swordfish and tuna provide a satisfactory vitamin D intake. Egg yolk, a few mushrooms and breakfast grains provide only a small intake of vitamin D that is nearly absent in meat and cheese.

Diet can be an important determinant of vitamin D status, and is influenced by the cultural nutritional practice and national policy [64]. Plasma 25(OH)D concentrations were lower in vegetarians and vegans than in meat and fish eaters in a UK study [65]. Consistently, the Hoorn Study in The Netherlands reported that the main determinants of vitamin D status were the time spent outdoors, the higher BMI, the consumption of oil-rich fish and fortified fat (fortified with 3 IU/g) and the use of vitamin D supplements [66].

In US, vitamin D is mainly ingested through fortified foods: milk and yogurt are the main contributors but other foods, such as breakfast grains, margarine, orange juice, and soy drinks, are also fortified. A report from NHANES showed that 100% of the population after two years of age had a vitamin D intake below the estimated average requirement and that this figure decreased by 7% with fortified foods and by 30% with additional supplements [67].

The dietary contribution to the desirable plasma levels of 25(OH)D is considerably lower in Italy than in US, due to the composition of diet (with less animal fats) and to the lack of appropriate fortification and supplementation of foods. In Italy, diet provides approximately 300 IU/day, so in Winter, when sun exposure is virtually absent, supplements for 1200–2000 IU/day must be guaranteed [68].


**We suggest** not to consider the dietary sources as adequate for the achievement of an optimal vitamin D status in Italy.


### 4.2. What about the Sun Exposure for the Treatment of Vitamin D Deficiency?

The main source of vitamin D for human body is the action of sunlight. Data about sun exposure and vitamin D synthesis are inconsistent, as sunlight accounts for 30% according to IOM [69] and for 80% according to Holick [29]. The skin synthesis of vitamin D is self-limited by the production of inactive metabolites that prevents the risk of vitamin D intoxication even after excessive sun exposure. Notably, the efficiency of skin production of vitamin D was adaptively increased by depigmentation when dark skinned people migrated from Africa to northern latitudes. Presently it was reported that a dark skin could produce up to six-times less vitamin D than a pale skin under the same UV exposure [70,71].

Italian epidemiologic studies reported that 25(OH)D levels differed by near 40% between subjects with either a low or an average sun exposure, suggesting a 60–90% contribution of the sun exposure to vitamin D synthesis [68]. Aging is associated with a decrease in the time of sun exposure, in the area of the exposed surface, and in the efficiency of the skin production of vitamin D [72]. Furthermore, from November to March the intensity of UVB rays is insufficient for the conversion of 7-dehydro-cholesterol into cholecalciferol, right above and below the 33th parallel (including also Mediterranean Europe) [73].

In young adults, a Summer sun exposure (without sunscreen) of about 25% of body surface (face and arms) for 15 min twice or thrice a week is equivalent to an oral dose of 25 μg (1000 IU) of vitamin D [74]. So, Summer vacation at sea plus 30 min daily in open space can be considered as sufficient for an adequate production and do not require a screening for vitamin D deficiency.

Workers without adequate sun exposure, such as indoor workers and rotating shift-workers mainly with night shift (included health professionals), should be added to the population traditionally considered at risk for vitamin D deficiency (see Section 3.1) [75].


**We suggest** not to consider sun exposure as adequate for the achievement of an optimal vitamin D status in Italy.


### 4.3. How to Supply Vitamin D?

Vitamin D is absorbed through passive diffusion and an incompletely known process involving membrane carriers, especially cholesterol transporters. Concomitant fat ingestion may improve vitamin D absorption [76], but vitamin D can be absorbed also without fat or oily vehicles. Factors that modify cholesterol absorption diminish vitamin D absorption as well.

There are conflicting data as to whether vitamin D2 and vitamin D3 are equally effective in increasing and maintaining serum concentrations of 25OH-D, particularly when a low dose treatment is performed. Several studies suggest that vitamin D3 should be preferentially used to optimize vitamin D status in the general population [77,78,79], in particular for the maintenance of adequate plasma 25(OH)D levels in the long term [80,81].

Notably, the oral assumption of vitamin D, either D2 or D3, appears more effective for increasing serum 25(OH)D than the equivalent dose given by injection both in the short and long-term assumption [82,83]. So, cholecalciferol, currently the most used therapy for the treatment of osteopenia/osteoporosis, should be used as first line therapy [6]. However, some vegan people may prefer the use of ergocalciferol that is not of animal origin [84].

Cholecalciferol is commercially available as drops (10,000 units/mL) and as vials with different potency (25,000 U, 50,000 U, 100,000 U, and 300,000 U) to be administered either orally or parenterally. No tablets are available in Italy.


**We recommend** treatment with cholecalciferol by mouth as the first line therapy in most patients.


### 4.4. What Is the Appropriate Dosage of Vitamin D Supplementation?

Vitamin D dosage and schedule depend on different factors: severity of deficiency, body weight, age of the patient, and need of rapid normalization of blood levels. It is usually appropriate to achieve target levels within 2–3 months [68,85] and, when the intestinal absorption is normal and baseline 25(OH)D levels are very low, in a healthy adult subject it has been estimated an average 0.7–1.0 ng/mL (1.7–2.5 nmol/L) rise for every 100 IU of daily ingested vitamin D [68]. Subsequently, the increase slows as the 25(OH)D levels rise.

When malabsorption is suspected, after performing what suggested at Section 3.4, the use of hydroxylated metabolites or injectable formulations of vitamin D may be considered.

The role of vitamin D supplementation in the prevention of falls is still controversial. A recent RCT [86] evaluated 200 elderly women, selected on the basis of a prior fall, divided into three groups on the basis of different monthly vitamin D3 dosage: 24,000 IU/monthly (control group), 60,000 IU/monthly or 24,000 IU/monthly plus 300 μg calcifediol. Although higher doses of supplements were more effective in reaching target levels of 25OH-D, the risk of falls was significantly increased. The study showed a 5.5 times greater risk of falling in patients reaching the highest quartile of 25(OH)D level (44.7–98.9 ng/mL) compared with those reaching the lowest quartile (21.3–30.3 ng/mL), suggesting an U-shaped (rather than a J-shaped) curve of the effect of vitamin D status on prevention of falls. A second RCT confirmed these data [87] but the association of the risk of falls and fractures with vitamin D status may be influenced by factors, such as assay standardization, lifestyle, or hypovitaminosis D-related disease masked by self-supplementation started before baseline vitamin D status assessment [88]. Finally, a RCT on community-dwelling old women showed a correlation between the annual oral administration of a large dose of cholecalciferol (500,000 IU) for 3–5 years and an increased the risk of falls [89].

A meta-analysis of 32 studies [90] revealed no association of serum 25(OH)D levels with all-cause mortality, while serum 25(OH)D levels were inversely associated with a lower all-cause mortality rates for values up to 70 ng/mL (175 nmol/L). A recent study showed that, after the standardization of the different assays for 25(OH)D levels, the risk of death from all causes increased with decreasing 25(OH)D levels < 16 ng/mL (40 nmol/L). No association was present for values between 16 ng/mL (40 nmol/L) and 48 ng/mL (120 nmol/L) [91].

Vitamin D deficiency and insufficiency are rapidly corrected by 50,000 IU of vitamin D once a week for 8 weeks [92,93]. A daily dose of 5000 IU of vitamin D for 8 weeks is an alternative approach [94]. Finally, to maintain vitamin D sufficiency, a simple strategy is the administration of 50,000 IU twice a month [84], or, alternatively, a daily dose of 1500–2000 IU [95].

The achievement of target 25(OH)D levels is not changed by different timing of vitamin D administration in controlled settings [78,96], while the adherence to treatment may be variable according to the vitamin D schedule (daily, weekly or monthly intervals). A deferred administration of dietary supplements may be useful to obtain adherence to the therapy and more stable 25(OH)D blood levels [97].


**We suggest** the following schedules for vitamin D supplementation:
Deficiency and insufficiency: 50,000 IU once a week for 8 weeks; alternatively, a daily dose of 5000 IU for 8 weeks;Maintenance of sufficiency: 50,000 IU twice a month; alternatively, a daily dose of 2000 IU.**We suggest** an individually tailored approach for vitamin D administration, involving the patient‘s opinion about the schedule (daily, weekly or monthly) that may offer the best adherence.


### 4.5. When Hydroxylated Metabolites of Vitamin D Should Be Prescribed?

**Calcifediol** has been reported to restore normal circulating levels of vitamin D more rapidly than cholecalciferol [98,99,100]. Reliable comparative evaluations of hydroxylated vitamin D metabolites vs. vitamin D-equivalent doses are lacking [101]. Calcifediol can be used in the general population and has an elective indication in congenital abnormalities of hepatic 25-hydroxylase activity [102], intestinal malabsorption and, sometimes, obesity [103].

Calcifediol is available in drops (0.15 mg/mL, where 1 drop contains 5 μg). Due to its potency, 3–4 drops/day or 20–30 drops/week of calcifediol are generally adequate to restore normal 25(OH)D plasma levels [98,99].

**Alpha-calcidiol and 1.25(OH)_2_D** (that is the mono- and di-hydroxylated vitamin D metabolites) should not be used for the routine treatment of vitamin D deficiency due to the risk of hypercalcemia and/or hypercalciuria and the unfeasibility of a reliable monitoring with plasma 25(OH)D levels [104]. On the other hand, in patients with chronic renal failure (CRF) the 1α-hydroxylation of vitamin D precursors is compromised [105]. So, the prevention of hypocalcemia, of secondary hyperparathyroidism, and of renal osteodystrophy requires the use of the active metabolite of vitamin D. The treatment of these patients is traditionally based on the administration of 1.25(OH)_2_D but in subjects with CRF is also present a 25(OH)D deficiency, due to nutritional factors, poor exposure to sunlight, and inhibition of liver 25-hydroxylation by uremic toxins [105]. Thus, the administration of cholecalciferol in post-dialytic phase contributes to the reduction of PTH levels [106]. The “Kidney Disease: Improving Global Outcomes” (KDIGO) guideline [105] suggests the measurement of 25(OH)D levels in patients with CRF and their correction according to the criteria used for the general population, in addition to appropriate changes in calcium and phosphate intake [107,108].

As PTH stimulates the renal 1-hydroxylation of 25(OH)D [1], in patients with hypoparathyroidism the activation of vitamin D is impaired. Thus, the treatment of hypoparathyroidism should be based on the use of the active form of vitamin D, i.e., 1.25(OH)_2_D [109].

Cholecalciferol has no direct action on bones, but patients with CRF [110,111] or hypoparathyroidism [109] with demonstrated vitamin D deficiency should be supplemented with cholecalciferol to warrant the “non-classical” effects of this vitamin.

1.25(OH)_2_D is commercially available as tablets (0.25 μg and 0.50 μg) and vials (1 μg/mL). Alpha-calcidiol is commercially available as tablets (0.25 μg and 1 μg) and drops (2 μg/mL, where 1 drop contains 0.05 μg).


**We suggest** the use of calcifediol in case of:
Hepatic impairment;Congenital abnormalities of the hepatic 25-hydroxylase enzyme;Malabsorption of cholecalciferol;Obesity.**We recommend against** routine use of 1.25(OH)_2_D or alpha-calcidiol for vitamin D deficiency.**We recommend** to use 1.25(OH)_2_D or alpha-calcidiol only when treating:
Chronic renal failure;Hypoparathyroidism.**We suggest** to use cholecalciferol as add on to 1.25(OH)_2_D, or alpha-calcidiol, in patients with CRF or hypoparathyroidism associated with demonstrated vitamin D deficiency.


### 4.6. Calcium Supplementation in Addition to Vitamin D in Osteoporotic Patients: Always, Never or Sometimes?

Calcium supplements in subjects with normal food intake are reported to increase the risk of nephrolithiasis and cardio- and cerebrovascular events [112,113,114]. As these data are still controversial [115,116,117,118], calcium supplements (at the dosage recommended for the different periods of life) should be prescribed only in case of documented nutritional deficiency [119].


**We recommend** calcium plus vitamin D supplements in patients with insufficient calcium intake, particularly if osteoporotic and taking bone active drugs.


### 4.7. Vitamin D Overtreatment: Myth or Reality?

The USA Endocrine Society guidelines recommend an upper threshold for daily vitamin D intake of 10,000 IU [6], while the IOM committee recommends an upper daily intake of 4000 IU [16]. However, clinical vitamin D toxicity is rare and blood concentrations of 25(OH)D associated with doubtless toxicity are rarely found [6]. No harm has been reported with a daily intake of 10,000 IU (250 μg) of vitamin D [1], while long-term studies on the effects of a daily intake greater than 10,000 IU or the maintenance of serum 25(OH)D levels above 100 ng/mL (250 nmol/L) are lacking. So, symptoms of vitamin D toxicity are unusual with a daily intake up to 10,000 IU, while toxicity may be associated with a daily intake > 10,000 IU [68]. Acute vitamin D toxicity, characterized by hypercalcemia and its associated symptoms, is generally reported in presence of serum 25(OH)D values > 140 ng/mL (350 nmol/L) [120,121,122].


**We recommend** a dosage of vitamin D up to a maximum of 4000 IU/day.**We recommend against** doses above 10,000 IU/day.**We suggest** a careful surveillance of any possible intake, because patients might inadvertently assume products containing additional amounts of vitamin D.


## 5. Treatment Monitoring

### 5.1. Vitamin D Assessment during Supplementation: When and How?

Vitamin D pharmacokinetics is complex and serum 25(OH)D level is influenced not only by vitamin D supplement but also by dietary vitamin D intake and exposure to sunlight [68,123].

Monitoring of serum 25(OH)D during supplementation is generally unnecessary but is appropriate in patients with symptomatic vitamin D deficiency, malabsorption conditions, and when poor compliance is suspected. In patients at risk of persistent 25(OH)D level below 30 ng/mL (75 nmol/L), retesting after 8–12 weeks may be appropriate. In the other patients, retesting should not be performed before 6 months of vitamin D supplementation [15,124,125,126].


**We recommend against** routine serum 25(OH)D testing during vitamin D supplementation.**We suggest** the assessment of vitamin D levels after at least 6 months of therapy, also if combined with bone active drugs, in patients:
With previous severe hypovitaminosis D or persistent risk of severe hypovitaminosis because of renal or liver failure, metabolic bone diseases, malabsorption, severe obesity, hypogonadism, glucocorticoid treatment;At risk for hypercalcemia due to underlying diseases (i.e., granulomatosis and lymphoproliferative tumors) where 1.25(OH)_2_D assay is appropriate for monitoring;Who assume high doses of vitamin D and present with symptoms of vitamin D toxicity.


### 5.2. Vitamin D and Drugs Interactions: What We Need to Know

The 25-hydroxylase CYP3A4 enzyme, which converts ergo- and cholecalciferol to 25(OH)D, is a phase I biotransformation enzyme for many drugs. Several drugs are metabolized by CYP3A4, while other medications may inhibit or induce CYP3A4 activity [127]. Further interfering mechanisms include:

- Altered absorption of the fat-soluble vitamin D with drugs that inhibit the absorption or enhance the elimination of dietary fat.
- Increased risk of hypercalcemia when vitamin D intake is associated with calcium-sparing medications.

Table 2 reports the drugs that may more frequently interfere with the absorption and metabolism of vitamin D.


**We suggest** the evaluation of concomitant medical treatments for a potential interference with vitamin D absorption and metabolism.**We suggest** the correction of vitamin D deficiency even in patients on teriparatide.


## 6. Special Contexts

### 6.1. Vitamin D and Pregnancy

Many pregnant women are at risk of vitamin D deficiency [6], a condition associated with increased risk of pregnancy complications, mainly pre-eclampsia and cesarean section [6]. A correlation between maternal vitamin D deficiency (<20 ng/mL, <50 nmol/L) and gestational diabetes, small for gestational age (SGA) newborns, preterm delivery, and pediatric asthma is reported [128,129]. Accordingly, these complications appear less frequent in pregnant women whose 25(OH)D levels are above 40 ng/mL (100 nmol/L) [128,130].

Vitamin D supplementation in pregnancy is safe up to 4000 IU/day [129]. A systematic review of RCTs demonstrated that prenatal vitamin D supplementation is associated with increased mean birth weight, reduced risk of SGA, reduced risk of wheeze in offspring, and increased infant length at one year of age, with no effect on preterm birth [131].

Supplementation should be individually tailored due to the variable response to treatment due to the different body weight and sun exposure [132].


**We suggest** to assay 25(OH)D levels in pregnancy to screen for its deficiency.**We suggest** the supplementation of pregnant women with cholecalciferol, aiming at a serum 25(OH)D level > 30 ng/mL (75 nmol/L).


### 6.2. BMI and Vitamin D Treatment: Does It Matter?

Obesity and vitamin D deficiency represent an important health problem worldwide, at least in western countries [133,134]. The relationship of serum 25(OH)D levels with BMI is controversial because both a negative and a positive correlation between these parameters, or its absence, were reported [135]. This variability may be explained by the cross-sectional design of most studies, but other variables may be relevant: latitude, season, gender (especially different adiposity between men and women with the same BMI) [136], dress customs [137], public health intervention on vitamin D supplementation [138], and living in developed or developing countries [139].

A well-conducted meta-analysis of 34 cross-sectional studies with adequate quality [135] demonstrated a weak, negative correlation between serum 25(OH)D levels and BMI in healthy adults, males and females, living in developed countries. The same correlation was evident also for men living in developing countries, but not for women. The strength of the meta-analyses, however, was low due to the high heterogeneity of the studies (*I*^2^ = 82.2%). Finally, a randomized study in obese subjects demonstrated that the 25(OH)D response to vitamin D3 supplementation is directly related to the dose and body size, with ≈2.5 IU/kg required for every unit (ng/mL) increment in 25(OH)D level [140].

In conclusion, a weak inverse correlation between serum vitamin D and BMI is demonstrated. Obese patients (BMI > 30 kg/m^2^) might require 2–3 times more vitamin D to both treat and prevent vitamin D deficiency and insufficiency [141]. Due to its pharmacokinetic profile, calcifediol might represent an alternative option [103,142,143,144].


**We suggest** to consider obese patients at high risk for vitamin D deficiency.**We suggest** a duplicated, or triplicated, dose of vitamin D in obese patients and the use of calcifediol instead of vitamin D in this setting.


## Figures and Tables

**Table 1:** Categories of patients that should be screened for vitamin D deficiency.

Osteomalacia Osteoporosis (particularly if bone active drugs are to be used) Older adults with history of falls Older adults with history of non-traumatic fractures Pregnant and lactating women Obese children and adults People not exposed to sufficient sun exposure Malabsorption syndromes (congenital or acquired) and bariatric surgery Chronic kidney disease Hepatic failure Cystic fibrosis Hyperparathyroidism Drug interfering with vitamin D metabolism (anti-seizure medications, glucocorticoids, AIDS medications, anti-fungals, cholestyramine) Granulomatous disorders and some lymphomas (in these cases, also 1.25(OH)_2_D should be tested)

**Table 2:** Vitamin D and drugs interaction.

Mechanism of Action	Drugs
Drugs that interfere with vitamin D absorption	Bile acid sequestrants (Cholestyramine) Lipase inhibitors (Orlistat)
Drugs that interfere with vitamin D metabolism	Antiepileptic drugs (phenobarbital, phenytoin)
	Corticosteroids
	Statins
	Antimicrobials (Rifampicin, Isoniazid, Hydroxychloroquine)
	Immunosuppressive agents (cyclosporine, tacrolimus)
	Chemotherapeutic agents
	Highly active antiretroviral agents
	Histamine H2-receptor antagonists
Drug-vitamin D interactions that may induce side effects	Thiazides

## Abbreviations

25(OH)D	25-hydroxy-vitamin D
ALP	Alkaline Phosphatase
AME	Italian Association of Clinical Endocrinologists
BMI	Body Mass Index
CRF	Chronic Kidney Disease
DXA	Dual energy X-ray Absorptiometry
FGF	Fibroblast Growth Factor
FRAX	Fracture Risk Assessment tool
GRADE	Grading of Recommendations, Assessment, Development, and Evaluation
IOM	Institute of Medicine
KDIGO	Kidney Disease: Improving Global Outcomes
LC	Liquid Chromatography
MS	Mass Spectrometry
LoE	Level of Evidence
NHANES	National Health and Nutrition Examination Survey
PTH	Parathyroid hormone
RANKL	Receptor Activator of Nuclear Factor-Kappa B Ligand
RCT	Randomized Clinical Trial
SD	Standard Deviation
SGA	Small for Gestational Age
US	Ultrasonography
VDBP	Vitamin D-Binding Protein
VDR	Vitamin D Receptor

## Appendix A

**Table A1:** The Level of Evidence of References.

Level of Evidence (LoE)	Reference Numbers
absence of evidence	[1,2,3,4,5,6,7,8], [10], [13], [16], [19], [20], [22], [24], [25], [29], [41,42,43,44], [46], [48], [50,51,52,53,54,55,56,57], [59,60,61,62,63], [67,68,69,70], [73], [74], [85], [88], [92], [93], [95], [97], [105,106,107,108,109,110], [119], [121], [123], [127,128,129], [133], [142,143,144]
very low (⊗◯◯◯)	[72], [76], [84], [126]
low (⊗⊗◯◯)	[15], [17], [18], [23], [28], [30], [32], [33], [38], [45], [58], [65], [66], [71], [75], [81,82,83], [91], [94], [101], [102], [120], [124], [136], [137]
moderate (⊗⊗⊗◯)	[9], [21], [26], [27], [31], [34,35,36,37], [39], [40], [49], [64], [77], [78], [90], [103], [116], [122], [134], [135], [139], [141]
high (⊗⊗⊗⊗)	[11], [12], [14], [47], [79], [80], [86], [87], [89], [96], [98,99,100], [104], [111,112,113,114,115], [117], [118], [125], [130,131,132], [138], [140]
